# Analysis and observation of the breakdown of Babinet’s principle in complementary spoof surface plasmon polariton structures

**DOI:** 10.1038/s41598-020-67923-5

**Published:** 2020-07-03

**Authors:** Go Itami, Osamu Sakai

**Affiliations:** Department of Electronic Systems Engineering, The University of Shiga Prefecture, 2500 Hassaka-cho, Hikone, Shiga 522-8533 Japan

**Keywords:** Microwave photonics, Surfaces, interfaces and thin films

## Abstract

A metal plate array (MPA) which is a structure complimentary to a metal hole array (MHA), supports spoof surface plasmon polaritons (SSPP) as well as an MHA does. Babinet’s principle attributes the phenomenon of duality to transmission characteristics of the complimentary impedance surfaces because of the symmetry of the behaviors of electric and magnetic fields. However, it is also a fact that the complimentary structures do not follow this principle if they have wavelength-size thickness, because electromagnetic waves do not treat such thick structures as a boundary surface but as propagation spaces with the specific boundaries such as a waveguide which shows SSPP modes. If the thickness is so small that it is negligible, Babinet’s principle is still valid, while it has been uncertain how the layer thickness works to break the principle as it is increased. The unconfirmed transformation is revealed analytically and experimentally with the use of MPAs and MHAs of varying thicknesses.

## Introduction

The behavior of electromagnetic waves interacting with objects is broadly classified into two cases. For wavelengths significantly larger than the object, the wave’s electric fields are treated as near fields and electromagnetic behavior is expressed as a scattering phenomenon. For wavelengths much smaller than the object the wave’s electric fields are treated as far fields and the electromagnetic behavior is expressed as propagating waves, either transmitted or reflected. However, when the wavelength is of the same order as the object size, the scattering phenomena become more complex. In this case, if such objects are perforated periodically, they can be provided an interesting medium with properties, otherwise not attainable with naturally occurring materials, such as a negative refractive index (NRI)^1–6^. In general, these artificial structures are called metamaterials.

In 2000, Pendry showed theoretically the concept of a perfect lens with a negative refractive index (NRI) medium^1^, and in 2001, Shelby et al. verified the possibility of an NRI experimentally by using the two artificial resonators for negative permeability and permittivity media^2^. Their studies demonstrated the feasibility of metamaterials, and accelerated the advances in the field in recent years^1–3, 7–12^. In such studies, a split ring resonator (SRR) is often used as a negative permeability medium for NRI realization. And the complementary split ring resonator (CSRR) is also known as the resonator for negative permittivity realization, based on Babinet’s principle^12–16^. In these two resonators, the essential difference in their resonance is based on the following electromagnetic radiation mechanisms: SRR radiates the topologically overlaid waves from the infinitesimal electric dipoles, and CSRR radiates them from the infinitesimal magnetic dipoles. Based on this principle, the SRR and CSRR show band-stop and band-pass characteristics if they are used as planar spatial filters.

A frequency selective surface (FSS) is also a spatial filter which has a two-dimensional structure with a unit cell and resonators, and is a metamaterial in a broader definition of the term^11,17–22^. Its operating principle is often expressed by using an equivalent circuit model. For example, a patch-type FSS and a slot-type FSS respectively show band-stop and band-pass characteristics, since they have series and parallel resonant circuits in their boundaries. Therefore, an FSS’s operation principle is also consistent with Babinet’s principle. On the other hand, a spoof surface plasmon polariton (SSPP) is also a feature of metamaterials^23–35^. It is the collective oscillation of electric fields like a surface wave in the microwave-frequency range. A metal hole array (MHA), which is a two dimensional conductive structure with periodic perforations, is often used for SSPP excitations^24–29^. When SSPP modes are excited on the surface, transmittance is up to 100 percent (extraordinary transmission)^36,37^ in an optimal frequency band, since the modes of both sides are coupled to each other.

From a different point of view, an MHA can be considered a spatial filter with band-pass effects. According to the SSPP theorem proposed by Pendry et al.^23, 29^, an MHA can be a macroscopic medium with a controllable permittivity of Drude-type frequency response, as is derived by solving a wave equation with microscopic boundary conditions such as waveguide modes taken into consideration. In the theorem, the boundary conditions are not limited to specific conditions^13,31,33^. For example, a metal plate array (MPA), which is the complementary structure of an MHA, should also be an SSPP-structure with band-pass effects.

However, an MPA is inversely treated as a band-stop FSS when it is a very thin structure. Although conventional studies have discussed clarifications of the relationship between SSPP modes and the structural parameters or the boundary conditions including duality^38^, the relevant part of an SSPP theory in terms of structural thickness in structure has not been discussed in detail. In other words, an MPA has the potential to provide evidence of the breakdown of Babinet’s principle.

In this study, an example of such a breakdown of Babinet’s principle is demonstrated by using an MPA and an MHA with thicknesses nearly the size of the interacting wavelength. First, changes in the relationships between electromagnetic behavior at the boundary conditions and the transmission or reflection properties of the two complementary structures, with the variations in their thickness, are discussed theoretically and analytically. The theoretical wave propagation model and the analytical study of MPAs indicated the possibility of a Babinet’s principle exception. And experimental results verified the theoretical assumption and analytical results using fabricated MPAs and MHAs with their wavelength-size thickness. This fact gives us more detailed physical insight into electromagnetic wave-propagations, and is useful for designing spatial filters, antennas and artificial lenses^20,39–42^.

In Section "Basis of spoof surface plasmon polaritons generations on a metal plate array", SSPP mode generation on an MPA is discussed by introducing a theoretical wave propagation model, and the effective relative permittivity and the dispersion relation are derived. In Section “Discussion of numerical results comparing metal plate array with metal hole array in frequency dependencies”, the changes in transmission and reflection properties and the angular dependencies of transmission characteristics of MPAs and MHAs are examined by varying their thickness analytically with the use of an electromagnetic simulator (HFSS, Ansys). In Section “Experimental demonstration of the breakdown of Babinet’s principle using metal plate arrays and metal hole arrays”, the transmission characteristics of the two structures in their thin and thick cases are examined experimentally. Finally, we summarize our study, which theoretically and experimentally demonstrates the breakdown of Babinet’s principle.

## Basis of spoof surface plasmon polaritons generations on a metal plate array

**Figure 1:**
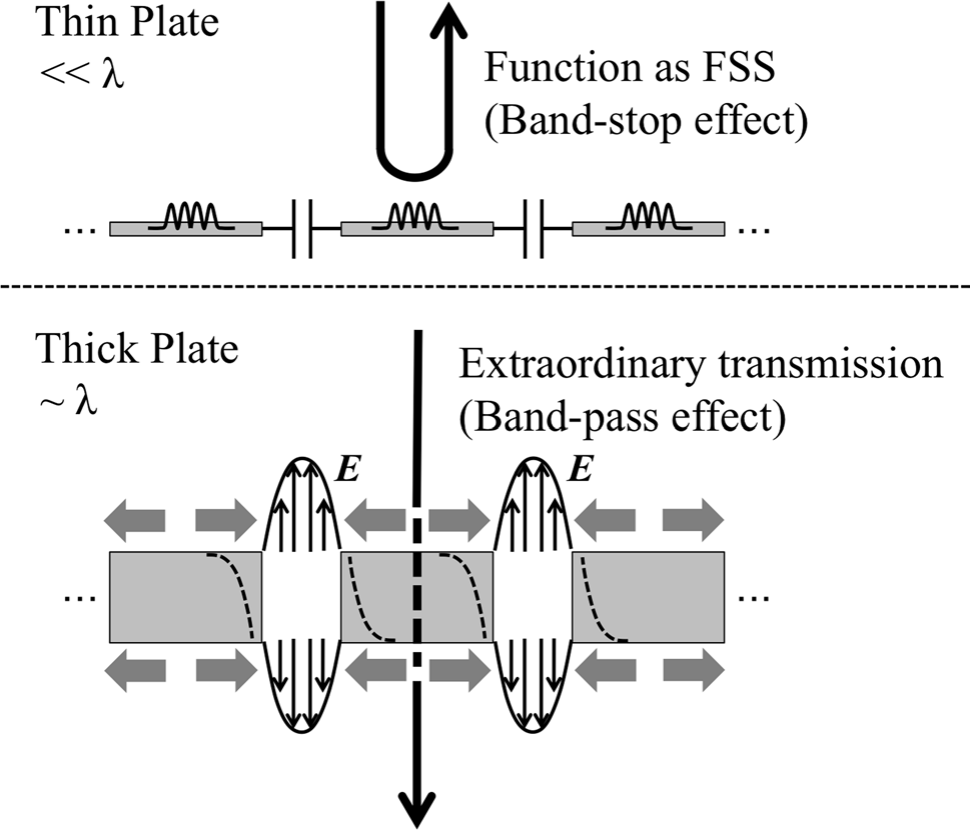
Physical concept of changing a propagation model in accordance with the boundary shift from an FSS-boundary to an SSPP-boundary in the case of a metal plate array.

In this study, the breakdown of Babinet’s principle is discussed based on the physical insights of SSPP mode generation and FSS’s operating principles (equivalent circuit model) ^17–19,21^. The phenomenon can be observed in an MPA by increasing its thickness, since the theoretical model of electromagnetic behavior on the boundary is changed from the latter to the former. If the MPA is a thin structure like a surface, its frequency characteristics can be expressed by using its equivalent circuit model, which shows a series resonant circuit in the admittance of an MPA. Also, if the MPA is a thick structure, its frequency characteristics can be expressed by using the SSPP theory proposed by Pendry et al.^23,29^, as shown in Fig. 1.

**Figure 2:**
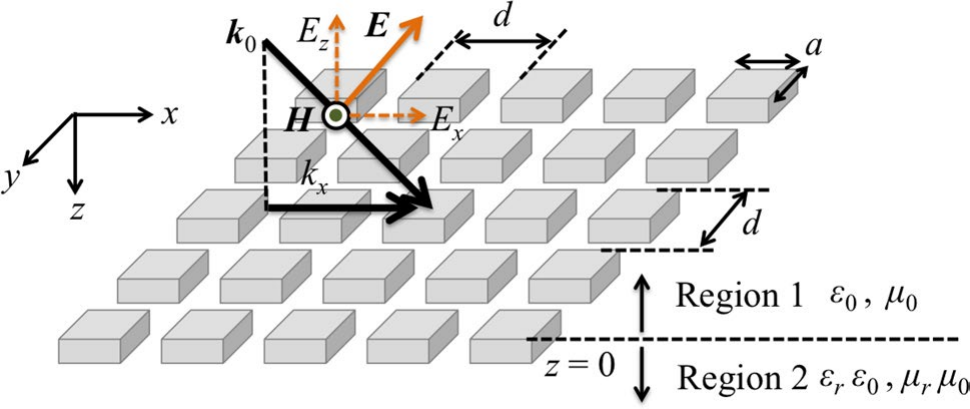
Schematic view of electromagnetic propagation on a metal plate array.

**Figure 3:**
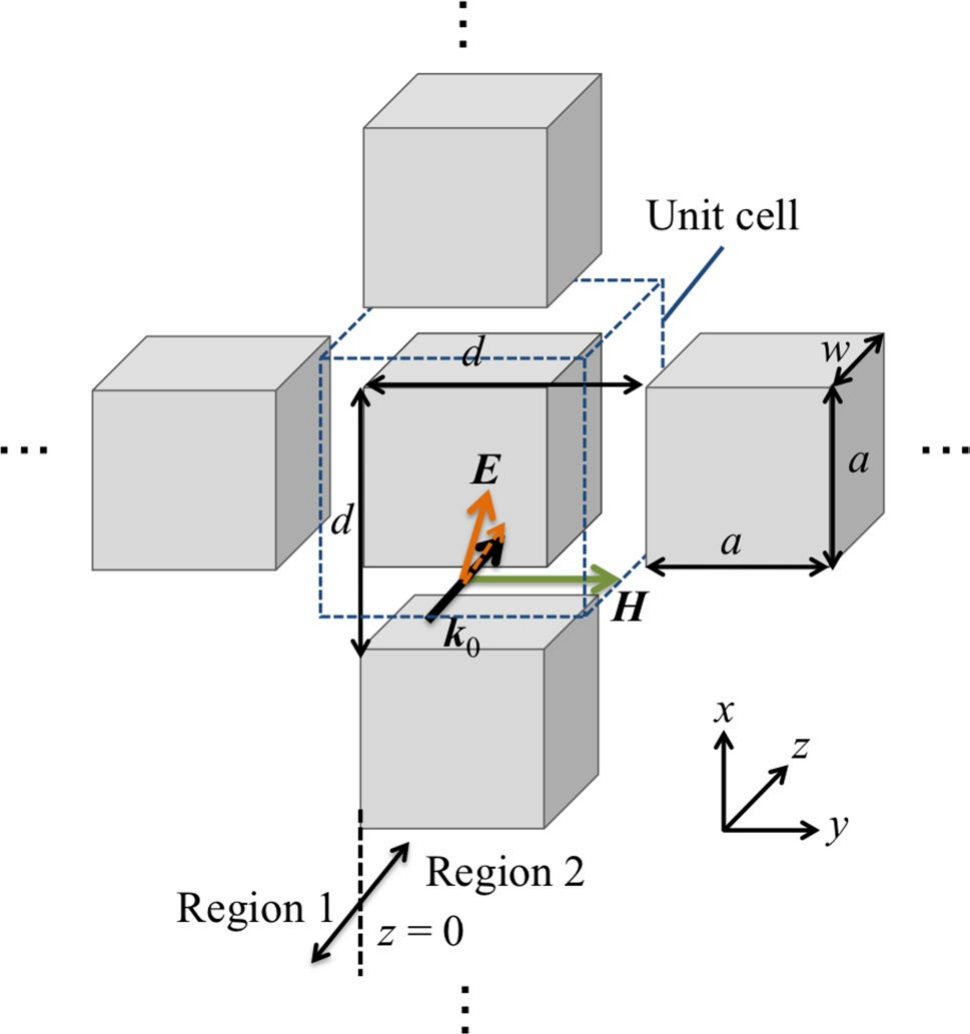
Microscopic view of the propagation on a metal plate array per unit cell.

Therefore, as well as SSPP mode generation on an MHA, an MPA can also generate SSPP modes on itself. The theoretical derivation can be described using the same framework. Here, TM-polarized waves entered an MPA at an oblique incidence as shown in Fig. 2, and each conductive plate in the MPA is assumed to be a perfect electric conductor. In this case, since an incident magnetic field ${\varvec{H}}$ has only a ${ y}$-component, the vector is expressed as ${\varvec{H}} = [\mathrm{0}, { H_y }, \mathrm{0}]$. And the incident electric field vector must also have only ${ x}$- and ${ z}$-components, and ${\varvec{E}} = [{ E_x}, \mathrm{0 }, { E_z}]$. The conductive plates are arranged two-dimensionally with a period ${ d}$, and the size of each plate is ${ a} \times { a}$, with depth *w*. It is assumed that the values of ${ a}$, ${ d}$ and ${ w}$ are of the same order as the incident wavelength, and a unit cell is defined as a space ${ d} \times { d}$ so as to satisfy the condition that an conductive plate is centered in it, as shown in Fig. 3. Assuming the propagation is from Region 1 to Region 2 in Fig. 2, the waves should propagate with parallel plate-waveguide modes around the boundary ${ z} = \mathrm{0}$. Note that the permittivity and permeability in Region 1 are $\epsilon =\epsilon _{\mathrm{0}}, \mu =\mu _{\mathrm{0}}$, respectively and the two values in Region 2 are also expressed as $\epsilon =\epsilon _{\mathrm{r}} \epsilon _{\mathrm{0}}, \mu =\mu _{\mathrm{r}} \mu _{\mathrm{0}}$. Here $\epsilon _{\mathrm{r}}$ and $\mu _{\mathrm{r}}$ are effective relative permittivity and permeability in Region 2; those also represent the macroscopic electromagnetic profiles of an MPA.

**Figure 4:**
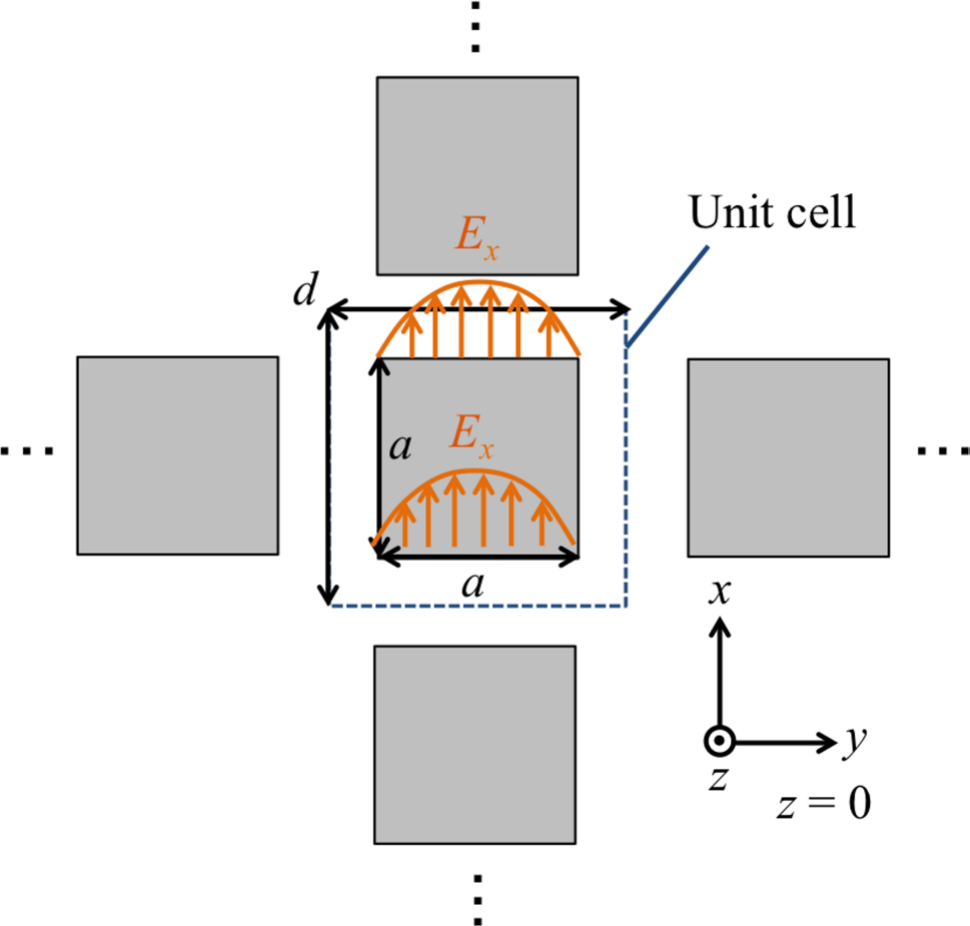
Electric distributions on a metal plate array following the microscopic boundary conditions.

Considering the boundary conditions, energy flow around the boundary, and an SSPP generation condition, macroscopic permittivity and permeability of an MPA are obtained, and an SSPP-dispersion relation in an MPA can be derived by using the two parameters. In the derivations, it is specified that $\mathrm{j} = \sqrt{\mathrm{-1}}$ and it is assumed that the incident wave is a plane wave; therefore the time operator is expressed as ${\partial }/{\partial t}$ replaced by $\mathrm{j} \omega$. Here, the ${ x}$- and ${ z}$-components of the incident electric field ${ E_x}$ and ${ E_z}$ in Region 1 are expressed as

1 $$\begin{aligned} E_{x}= & {} E_{x \mathrm{0}} \exp {\mathrm{j}(k_x x+ k_z z)}, \end{aligned}$$

2 $$\begin{aligned} E_{z}= & {} E_{z \mathrm{0}} \exp {\mathrm{j}(k_x x- k_z z)}. \end{aligned}$$

In the above expressions, the time vibrational term $\exp {(\mathrm{j}\omega t)}$ is omitted for simplicity. Note that $E_{x \mathrm{0}}$ and $E_{z \mathrm{0}}$ are the amplitudes values of $E_{x}$ and $E_{z}$, and that ${ k_x}$ and ${ k_z}$ are the ${ x}$- and ${ z}$-components of the incident wave vector ${\varvec{k}}$. On the other hand, considering the propagation modes inside an MPA, the waves should have parallel-plate waveguide modes. Here, if the incident wavelength satisfies ${\lambda }>{ a}$ and a miniaturized unit cell is used (${ a}>{ d}/\mathrm{2}$), the fundamental waveguide mode is dominant^23,29^. And in a unit cell of an MPA, the propagation modes can be formed, as shown in Fig. 4. Thus, the ${ x}$-components of the electric field $E_{x}$ can take the form

3 $$\begin{aligned} E_{x}= & {} E_{\mathrm{0}} \sin {\left( \frac{\pi y}{a}\right) } \exp {(-\beta z)}, \end{aligned}$$

4 $$\begin{aligned} \beta= & {} \sqrt{\left( \frac{\pi }{a}\right) ^{\mathrm{2}} - {\omega }^2 \epsilon _{\mathrm{0}} \mu _{\mathrm{0}} } \end{aligned}$$

Here, ${\beta }$ is a propagation constant, and $E_{x}$ shows the electric field of the waveguide mode. Note that the correspondence between the *E*-component (*x*) and the waveguide mode (*y*) is inversed if the incident condition rotates 90 degrees, and the wave does not depend on the propagation direction. The mode is excited on an MPA per unit cell as shown in Fig. 4. And other modes are cut off due to the two narrow boundaries. When an MPA is treated as a macroscopic medium, the average electric field intensity per unit cell can be derived as

5 $$\begin{aligned} \overline{E_{\mathrm{0}}}= & {} E_{\mathrm{0}} \frac{\mathrm{1}}{d^{\mathrm{2}}} (d-a) \int _{\mathrm{0}}^a \sin {\left( \frac{\pi y}{a}\right) } dy \nonumber \\= & {} \frac{\mathrm{2}a(d-a)}{\pi d^{\mathrm{2}}} {E_{\mathrm{0}}} \end{aligned}$$

Where $\overline{E_{\mathrm{0}}}$ is the average electric field intensity. Considering an energy flow across the boundary $z = \mathrm{0}$, the inflow from the upper side and the outflow to lower side must take the same value. Here, if an MPA is treated as a microscopic structure or macroscopic uniform medium, the energy flow can be expressed in two ways, that is

6 $$\begin{aligned} ({{\varvec{E}}} \times {{\varvec{H}}})_{z, \mathrm{micro}}= & {} \frac{-k_z E_{\mathrm{0}}^{\mathrm{2}}}{\omega \mu _{\mathrm{0}}} \frac{\mathrm{1}}{d^{\mathrm{2}}} (d-a) \int _{\mathrm{0}}^a \sin ^{\mathrm{2}}{\left( \frac{\pi y}{a}\right) } dy = \frac{-k_z}{\omega \mu _{\mathrm{0}}} {E_{\mathrm{0}}}^{\mathrm{2}} \frac{a(d-a)}{\mathrm{2}d^{\mathrm{2}}}, \end{aligned}$$

7 $$\begin{aligned} ({{\varvec{E}}} \times {{\varvec{H}}})_{z, \mathrm{macro}}= & {} \frac{-k_z}{\omega \mu _{\mathrm{r}} \mu _{\mathrm{0}}} {\overline{E_{\mathrm{0}}}}^{\mathrm{2}} = \frac{-k_z}{\omega \mu _{\mathrm{r}} \mu _{\mathrm{0}}} {E_{\mathrm{0}}}^{\mathrm{2}} \frac{\mathrm{4} a^{\mathrm{2}}(d-a)^{\mathrm{2}}}{\pi ^{\mathrm{2}} d^{\mathrm{4}}}. \end{aligned}$$

And considering the equation $({{\varvec{E}}} \times {{\varvec{H}}})_{z, \mathrm{micro}} = ({{\varvec{E}}} \times {{\varvec{H}}})_{z, \mathrm{macro}}$, the macroscopic relative permeability $\mu _{\mathrm{r}}$ is obtained as

8 $$\begin{aligned} \mu _{\mathrm{r}} = \frac{\mathrm{8} a(d-a)}{\pi ^{\mathrm{2}} d^{\mathrm{2}}}. \end{aligned}$$

The macroscopic relative permittivity $\epsilon _{\mathrm{r}}$ also can be obtained by considering the propagation constant, as well as the energy flow. If treating an MPA as a macroscopic medium, the waves in Region 2 cannot propagate in *x*- and *y*-directions. Therefore, the incident wave vector in the macroscopic medium should have only a *z*-component. In other words, the wave vector ${\varvec{k'}}$ can be expressed as ${\varvec{k'}}=[\mathrm{0}, \mathrm{0}, k'_z]$, and $k'_z$ is

9 $$\begin{aligned} k'_z = \sqrt{\epsilon _{\mathrm{r}} \mu _{\mathrm{r}}}k_{\mathrm{0}}. \end{aligned}$$

Here, $k_{\mathrm{0}}$ is the wavenumber in free space, and it satisfies $k_{\mathrm{0}}=\omega \sqrt{\epsilon _{\mathrm{0}} \mu _{\mathrm{0}} }$. Therefore the macroscopic relative permittivity $\epsilon _{\mathrm{r}}$ can be derived by using the following equation if considered that the propagation constant inside an MPA is treated as a microscopic or a macroscopic medium,

10 $$\begin{aligned} (k'_{z} =) \sqrt{\epsilon _{\mathrm{r}} \mu _{\mathrm{r}} } k_{\mathrm{0}} = \mathrm{j} \beta . \end{aligned}$$

Then, by substituting (4) and (8) into (10) and squaring the equation, the values of $\epsilon _{\mathrm{r}}$ are obtained as

11 $$\begin{aligned} \epsilon _{\mathrm{r} } = \frac{ \pi ^{\mathrm{2}} d^{\mathrm{2}} }{ \mathrm{8} a(d-a) } \left[ \mathrm{1}-\frac{(\pi c_{\mathrm{0}})^{\mathrm{2}} }{a^{\mathrm{2}}\omega ^{\mathrm{2}}}\right] . \end{aligned}$$

Note that $c_{\mathrm{0}}$ is the velocity of light in free space, and the equation (11) is usually expressed the following formula,

12 $$\begin{aligned} \epsilon _{\mathrm{r}} = \frac{ \pi ^{\mathrm{2}} d^{\mathrm{2}} }{ \mathrm{8} a(d-a) } \left[ \mathrm{1}-\frac{ {\omega _{\mathrm{p}}}^{\mathrm{2}} }{\omega ^{\mathrm{2}}}\right] , \ \ \ \omega _{\mathrm{p}} = \frac{\pi c_{\mathrm{0}}}{a}. \end{aligned}$$

Here, $\omega _{\mathrm{p}}$ is the cut-off frequency of an MPA. As shown in Equation (12), an MPA also has a relative permittivity with the same frequency response as an MHA^23^. This frequency-responsive dielectric medium is known as a Drude model, which implies the oscillation of free electrons in a good conductor. Therefore, the fact indicates that electric field vibrations in an MPA simulate plasmonic oscillations, so an MPA is also considered to be an SSPP-structure as well as an MHA. Based on the above discussion, the dispersion relation in an MPA is derived by assuming a surface wave formation on the boundary of an MPA, treated as a macroscopic medium. Since TM-polarized incident waves have only a *y*-component in the model, an amplitude with a *z*-dependency of $H_y$ on the boundary between Region 1 and Region 2 can be expressed by the following formula, with attenuation characteristics in each condition,

13 $$\begin{aligned} h(z) = {\left\{ \begin{array}{ll} h_{\mathrm{1}} \exp {(K_{\mathrm{1}}z)} &{} z<\mathrm{0} \\ h_{\mathrm{2}} \exp {(-K_{\mathrm{2}}z)} &{} z \ge \mathrm{0} \end{array}\right. }. \end{aligned}$$

Here $h_{\mathrm{1}}$ and $h_{\mathrm{2}}$ are amplitudes of *h*(*z*), and $K_{\mathrm{1}}$ and $K_{\mathrm{2}}$ are macroscopic propagation constants on the boundary. And by considering the wave equation of $H_y$, $K_{\mathrm{1}}$ and $K_{\mathrm{2}}$ can be derived as $K_{\mathrm{1}} = \sqrt{ {k'_x}^{\mathrm{2}} - {\omega }^{\mathrm{2}} \epsilon _{\mathrm{0}} \mu _{\mathrm{0}}}$, $K_{\mathrm{2}} = \sqrt{ - {\omega }^{\mathrm{2}} \epsilon _{\mathrm{r}} \mu _{\mathrm{r}} \epsilon _{\mathrm{0}} \mu _{\mathrm{0}} }$. In this derivation, it should be noted that since the waves cannot propagate in the *x*-direction when an MPA is treated as a macroscopic medium, the *x*-component of the wavenumber in Region 2 must be zero. And considering the boundary condition between Region 1 and Region 2, both tangential components of the magnetic fields take the same value at $z = \mathrm{0}$, and the same is true for the electric field. That is

14 $$\begin{aligned} K_{\mathrm{1}} = -\frac{ K_{\mathrm{2}} }{\epsilon _{\mathrm{r}} }, \ \ \ h_{\mathrm{1}} = h_{\mathrm{2}}. \end{aligned}$$

Equation (14) is often called the generation condition of surface plasmon polariton. With the use of the above definitions of $K_{\mathrm{1}}$ and $K_{\mathrm{2}}$ and the expressions (12) and (8), an SSPP-dispersion relation in an MPA is obtained by squaring Eq. (14),

15 $$\begin{aligned} {k_{\mathrm{||}}}^{\mathrm{2}} {c_{\mathrm{0}}}^{\mathrm{2}} = \omega ^{\mathrm{2}} + \frac{ \omega ^{\mathrm{4}}}{\omega _{\mathrm{p}}^{\mathrm{2}}-\omega ^{\mathrm{2}}} \frac{ \mathrm{64} a^{\mathrm{2}} {(d-a)}^{\mathrm{2}} }{ {\pi }^{\mathrm{4}} d^{\mathrm{4}} }. \end{aligned}$$

**Figure 5:**
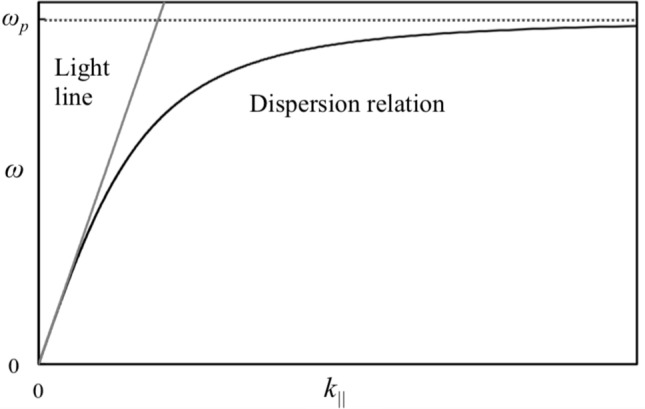
Ideal SSPP-dispersion relation in metal plate array.

Note that ${k'_x}$ is replaced by $k_{\mathrm{||}}$ in the derivation of Equation (15). The curve is shown in Figure 5, and it confirms the similarity of this curve and that of an MHA^23,29^. Therefore, an MPA has an ability of conserving SSPP-modes and shows the phenomenon of an extraordinary transmission as well as an MHA. Here, it is also known that this curve does not intersect with a light line since lattice scattering effects are not taken into account for the curve. If the effects are introduced into the curve, the wavenumber of $k_{\mathrm{||}}$ in it has to be replaced with

16 $$\begin{aligned} {k'_{\mathrm{||}}} = {k_{\mathrm{||}}} \pm n \mathrm{|} {{\varvec{G}}}_{x} \mathrm{|} \pm m \mathrm{|}{{\varvec{G}}}_{y} \mathrm{|}, \ \ \ \mathrm{|} {{\varvec{G}}}_{x} \mathrm{|} = \mathrm{|} {{\varvec{G}}}_{y} \mathrm{|} = \frac{\pi }{d}. \end{aligned}$$

**Figure 6:**
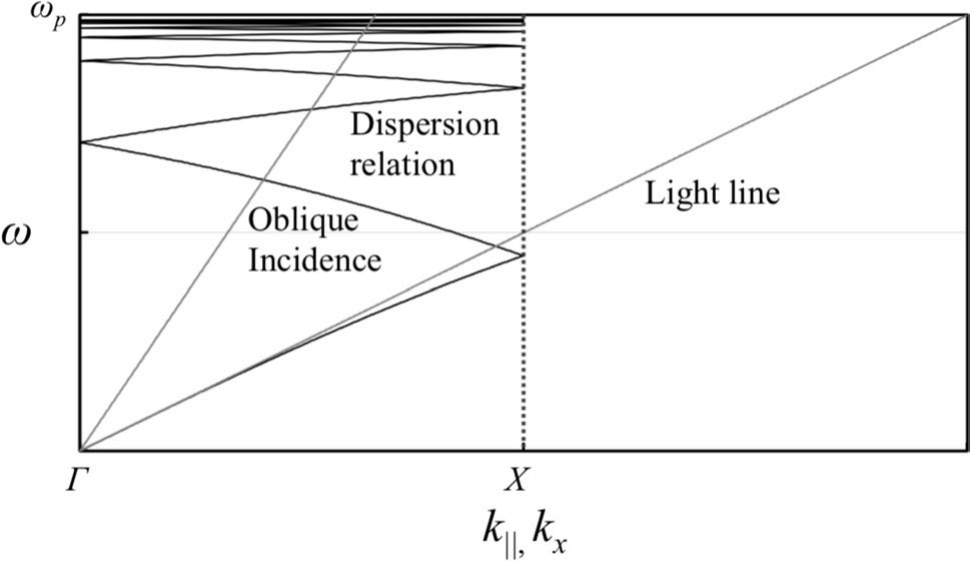
An actual SSPP-dispersion relation in a metal plate array and light line including the case of oblique incidence.

Note that ${k'_{\mathrm{||}}}$ is the wavenumber of an SSPP with scattering effects, *n* and *m* are positive integers, and ${{\varvec{G}}}_{x}$ and ${{\varvec{G}}}_{y}$ are the reciprocal lattice vectors in *x*-direction and *y*-direction respectively. The SSPP-dispersion relation in an MPA with the scattering effects is shown in Fig. 6, which shows that the curve intersects a light line at several points. SSPP-modes are formed at those points, and the frequencies are considered to be resonant frequencies of SSPPs. The dispersion relation also shows that the MPA has band-pass effects caused by extraordinary transmission as in the case of an MHA. Thus, an MPA acts as a band-pass filter if it is thick enough to be regarded as an SSPP-structure, and also acts as a band-stop filter, if the MPA is thin enough to be regarded as a kind of FSS. The above discussion supports the possibility that an MPA can be changed from a band-stop filter to a band-pass filter as its thickness is increased. This indicates that the breakdown of Babinet’s principle, since an MHA shows band-pass effects regardless of its thickness and an MPA is a complementary structure to an MHA.

## Discussion of numerical results comparing metal plate array with metal hole array in frequency dependencies

**Figure 7:**
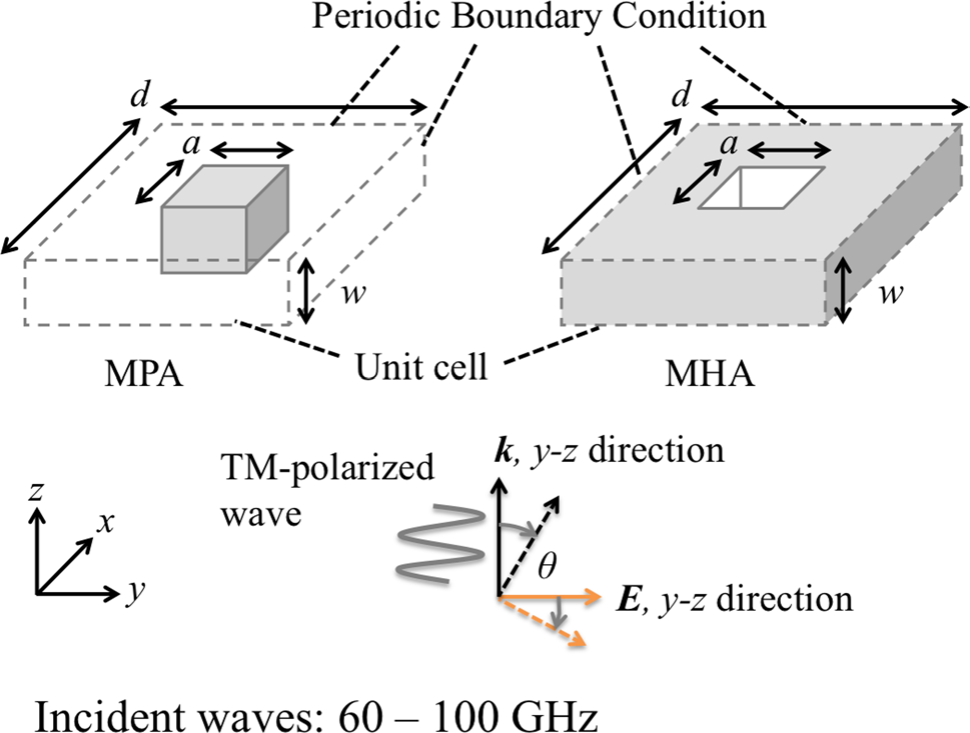
Analytical model of metal plate array and metal hole array for comparing their transmittances.

**Figure 8:**
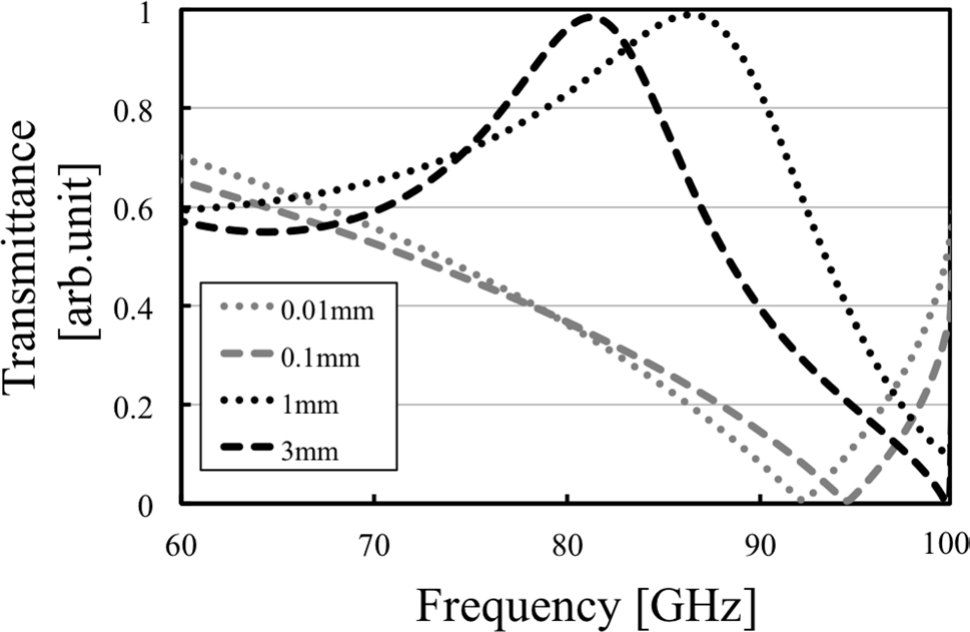
Thickness dependency of transmission characteristics in the case of the metal plate array.

**Figure 9:**
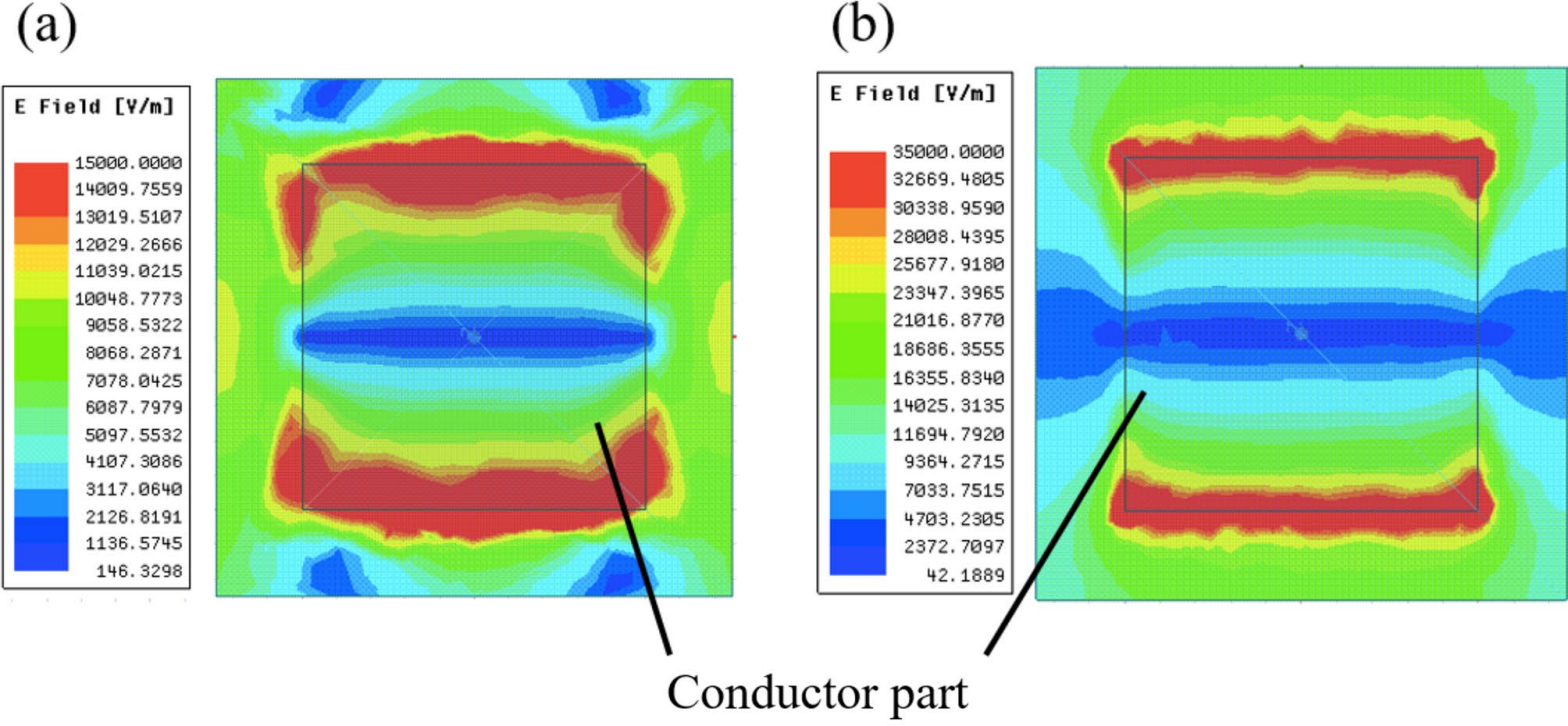
Electric distributions on MPAs at resonant frequencies in the cases of thickness 0.01 mm and 3 mm; (**a**) thickness is 0.01 mm and the resonant frequency is 92 GHz, (**b**) thickness is 3 mm and the resonant frequency is 81 GHz. In both cases, distributions are measured on the surface of the plates.

**Figure 10:**
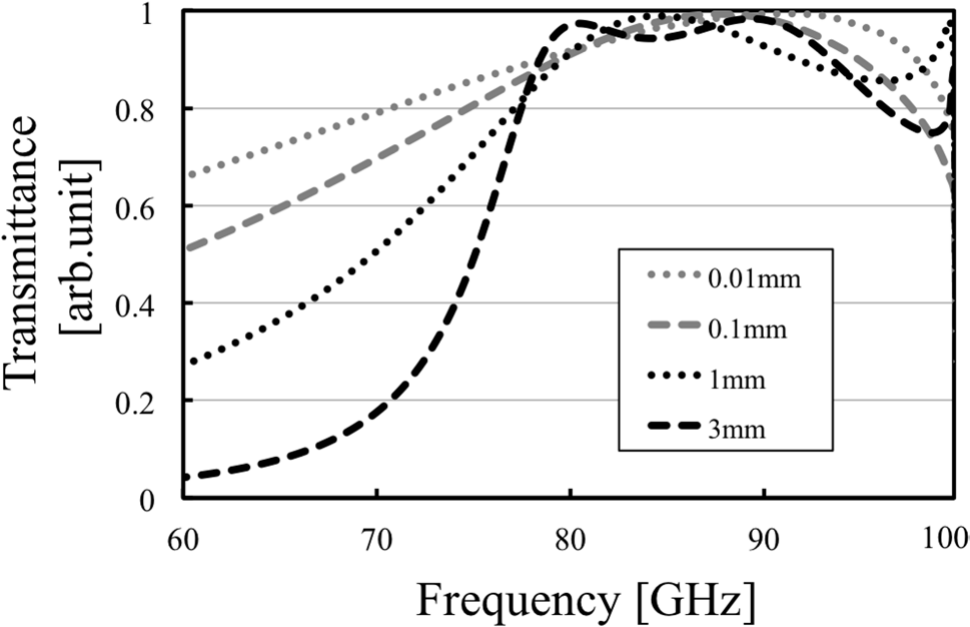
Thickness dependency of transmission characteristics in the case of the metal hole array.

**Figure 11:**
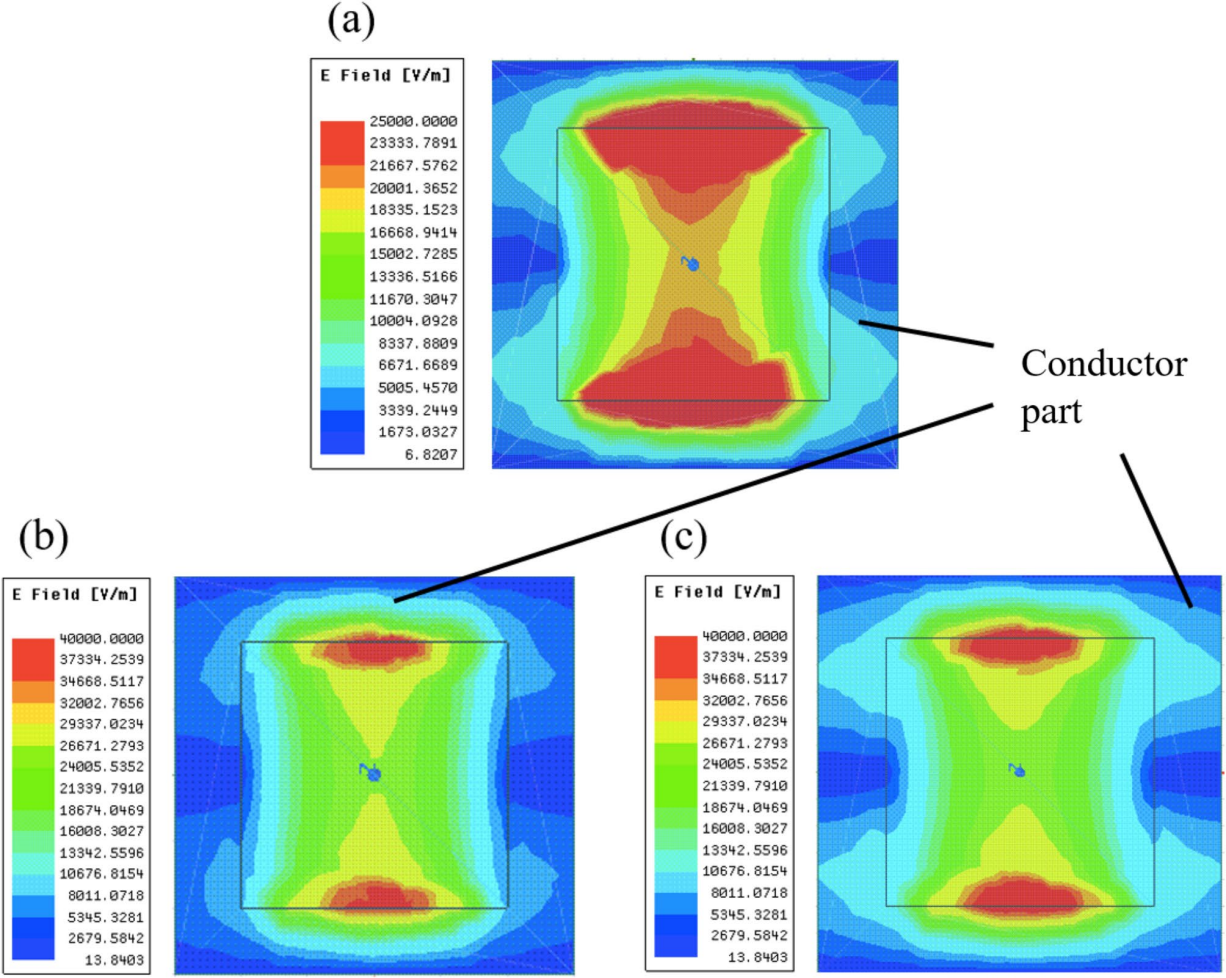
Electric distributions on MHAs at resonant frequencies in the cases of thickness 0.01 mm and 3 mm; (**a**) thickness is 0.01 mm and the resonant frequency is 84 GHz, (**b**) thickness is 3 mm and the resonant frequency is 80 GHz, (c): thickness is 3 mm and the resonant frequency is 90 GHz. In all the cases, distributions are measured on the surface of the plates.

In this section, the breakdown of Babinet’s principle is confirmed by using MPAs and MHAs in electromagnetic numerical analyses. According to the discussion in the previous section, the two complementary structures, MPAs and MHAs should show different tendencies in their electromagnetic profiles such as transmittance of incident waves if their thicknesses are varied sufficiently. That is, an MPA should show a change from band-stop effects to band-pass effects as its thickness increases; on the other hand, an MHA should consistently show band-pass effects regardless of its thickness. To examine the above conclusions, the two models of an MPA and an MHA are provided as shown in Fig. 7. Note that the size of the MPA’s unit cell is $a \times a \times w$ and the MPA unit cell is two-dimensionally arranged infinitely with a period *d*, and an MHA’s holes are centered in an unit cell with of size of $a \times a \times w$, and the MHA unit cell is two-dimensionally arranged infinitely with a period *d*. Based on the previous studies of MHA under the cut-off frequency^23,29,35^, waves of 60 - 100 GHz are introduced in the two models in a *y*-*z* direction, which has an incident angle $\theta$. Transmission characteristics of the two models are analyzed with variations of their thickness *w* in a range of 0.01 mm - 3 mm. Here, the analyses are conducted by using an electromagnetic simulator (HFSS R19, Ansys, Canonsburg, PA, USA) and the values of *a* and *d* are fixed as (*a*, *d*) = (2 mm, 3 mm) in the analyses. The transmittance results and electric distribution results at each resonant frequency for the MPA and the MHA are shown in Figs. 8, 9, 10, and 11, respectively.

The results in Fig. 8 confirm the change of transmission characteristics in the MPA from band-stop effects to band-pass effects. In the cases of 0.01 mm and 0.1 mm, the results show that the MPA possesses band-stop effects because the structure is much thinner than the incident wavelengths, and the MPA is considered to act as an FSS. On the other hand, in the case of 1 mm and 3 mm, the MPA possesses band-pass effects since the MPA has a thickness similar to the incident wavelengths and is considered to act as an SSPP-structure. The results in Fig. 9 also supports the difference of propagation models between the thin case (a) 0.01 mm and the thick case (b) 3 mm. Although the electric distribution spreads around the conductor part in the case (a), the distribution in the case (b) are concentrated only on the horizontal edges of the conductor. The distribution shows parallel plate-waveguide modes as proposed in Fig. 4. The results in Fig. 8 also show the shift of resonant frequencies in all the cases, with the reason considered to be related to the change of propagation models. Specifically, the shift in the two thick cases is larger than that in the thin cases, although the ratio of the increase in thickness in the thick cases is smaller than that in the thin cases, thus indicating that the frequency-shift is sensitive to the ratio of the thickness and the wavelength. From the discussion, the facts support the above assumption, since the shift is related to the MPA-thickness.

On the other hand, the results in Fig. 10 confirm that the MHA consistently shows the same transmission characteristics, with band-pass effects in all the cases. In the thin cases of 0.01 mm and 0.1 mm, the MHA shows band-pass effects, as it is considered to act as an FSS with parallel resonant circuits^19,21^. Also, in the thick cases of 1 mm and 3 mm, the MHA shows band-pass effects, as it is considered to act as an SSPP-structure. Specifically, the result in the thick case of 3 mm shows almost perfect transmittance in the two bands, while other results do not show this phenomenon. The reason is considered to be related to the change of the propagation model in the MHA because if it acts as an FSS, its geometric configuration cannot have such characteristics in the adjacent frequency bands. In contrast, if it acts as an SSPP-structure, its geometric configuration can possess such characteristics in the adjacent frequency bands because the intersection of the light line and the SSPP-dispersion relation appears at multiple points, which indicate resonant conditions. Although these points are not certain to be observed in real events, the SSPP structure has the potential of having multiple resonant frequencies. The results of electric distributions in Fig. 11 also shows the differences of resonant mechanisms between FSS and SSPP modes. In the thin case (a) 0.01 mm, it finds that the distributions spread around the two horizontal edges which is considered to work as a capacitor. On the other hand, the distributions in the thick cases (b) and (c) 3 mm are concentrated on the center of the two edges since they form parallel plate-waveguide modes. From the discussions, the facts support the above assumption since the thin cases show capacitor effects and one of the thick cases shows SSPP modes. And in a viewpoint of the breakpoint of Babinet’s principle, the two results of the thickness 0.01 mm do not break the principle since the results show the same resonant frequency of 92 GHz with different band effects. However, in the cases of 0.1 mm, the resonant frequency of the MPA is shifted from that in the case of 0.01 mm and the resonant frequency of MPA differs from that of the MHA. Therefore, the principle is considered to be broken around the thickness 0.1 mm in the models.This thickness corresponds to the thickness-wavelength ratio of 0.03 and the phase shift of 0.19 rad.

**Figure 12:**
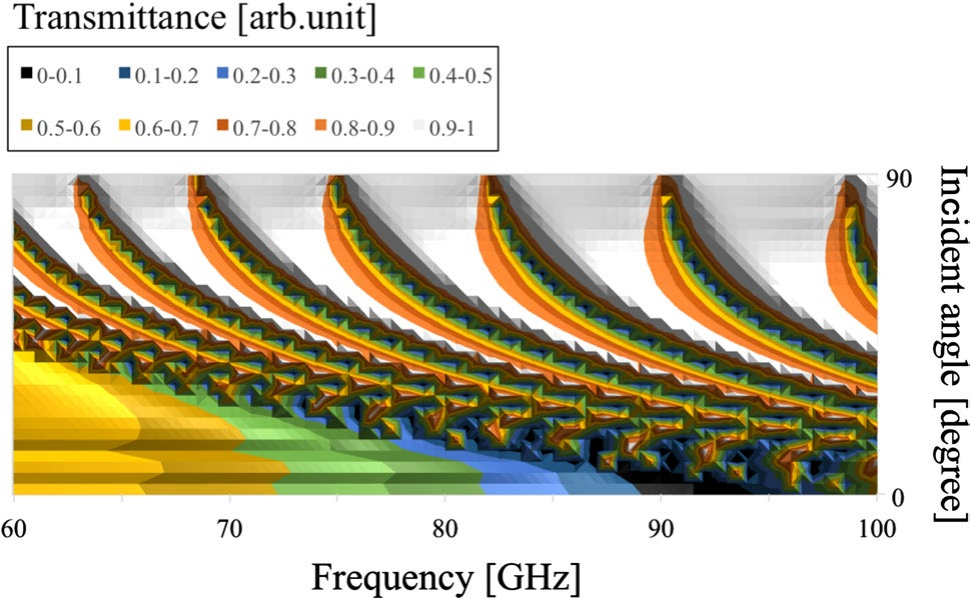
Angular dependency of transmission characteristics in the case of the metal plate array with a thickness of 0.01 mm.

**Figure 13:**
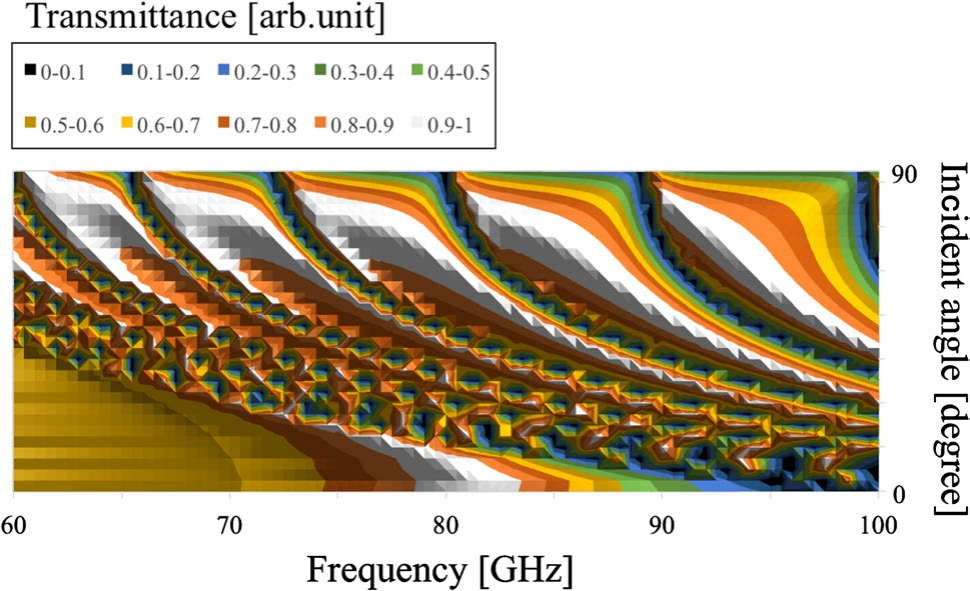
Angular dependency of transmission characteristics in the case of the metal plate array with a thickness of 3 mm.

**Figure 14:**
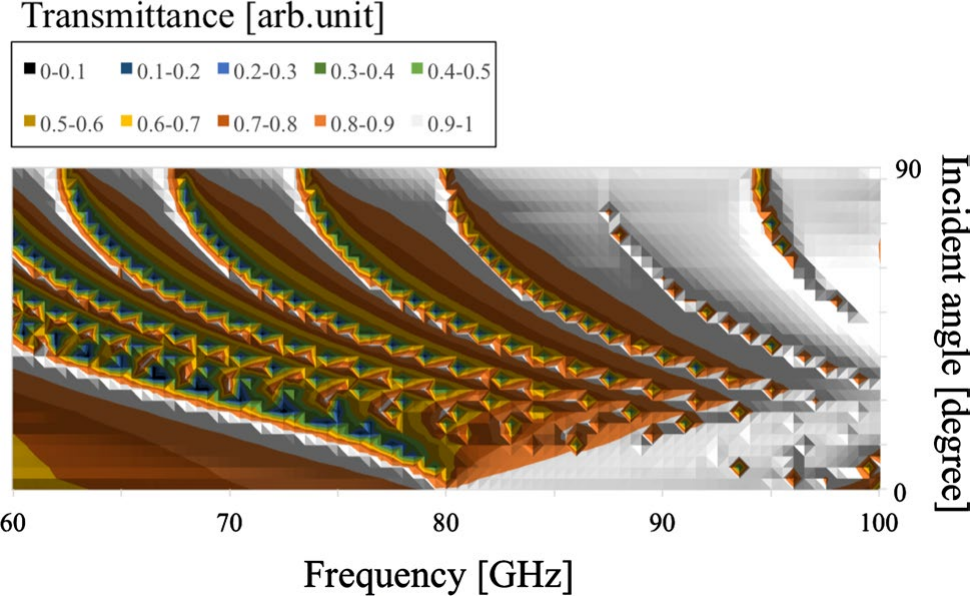
Angular dependency of transmission characteristics in the case of the metal hole array with a thickness of 0.01 mm.

**Figure 15:**
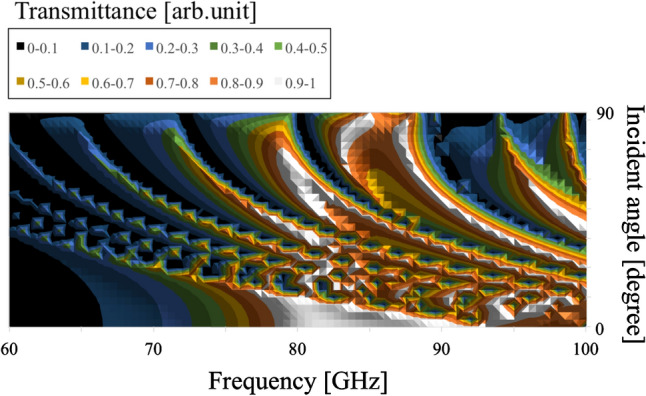
Angular dependency of transmission characteristics in the case of the metal hole array with a thickness of 3 mm.

Next, the angular dependency of their transmission characteristics is investigated by varying values of $\theta$ and their thickness, in the thin (0.01 mm )and thick (3 mm) cases. The results in the four cases are shown in Figs. 12, 13, 14, and 15, respectively.

In Figs. 12 and 13, the two results confirm that both MPAs have several band-gap modes that vary in accordance with incident angles. However, the behavior of these modes differs; for example, there is no frequency distribution of the transmittance between the adjacent band gaps in the thin case (Fig. 12). On the other hand, there are frequency distributions of the transmittance between the adjacent band gaps in the thick case (Fig. 13). These facts indicate different propagation modes in the two cases. Furthermore, comparing the two frequency characteristics at around 90 degrees, it is found that there are strong transmission regions in the thin cases although there are almost no strong transmission regions in the thick case. The reason is considered to be that in the thin case, propagation waves treat the MPA as a boundary so that transmission waves are formed by each scattered wave from each unit cell though incident waves have no vertical components of the wave vector. In contrast, in the thick case, since propagation waves treat the MPA as periodically arranged waveguides, transmission waves are considered effectively not to be formed, as incident waves hardly have any vertical components of their wave vector.

In Figs. 14 and 15, as well as the results for the MPAs, these results also confirm the difference of frequency distribution of the transmittance between the adjacent modes of the two angular dependencies. This fact shows the difference of the propagation modes between the thin case (Fig. 14) and thick case (Fig. 15). However, it can be seen that the modes in the thick case are band-pass modes, while the modes in the thin case are band-gap modes. This result shows the characteristics of propagation modes in a square waveguide which has limited higher-order modes, although a parallel plate waveguide has unlimited higher modes. In other words, the difference of the modes between the thick MPA and the thick MHA originates in the difference of transmission-band percentage in the entire frequency band. In the discussions, the differences of the propagation modes between the thin and thick cases in both structures are confirmed for the two structures. These results indicate that the modes in the thin cases are determined by the boundary with frequency responses and that those in the thick cases are supported by the waveguide modes of MPAs and MHAs.

## Experimental demonstration of the breakdown of Babinet’s principle using metal plate arrays and metal hole arrays

**Figure 16:**
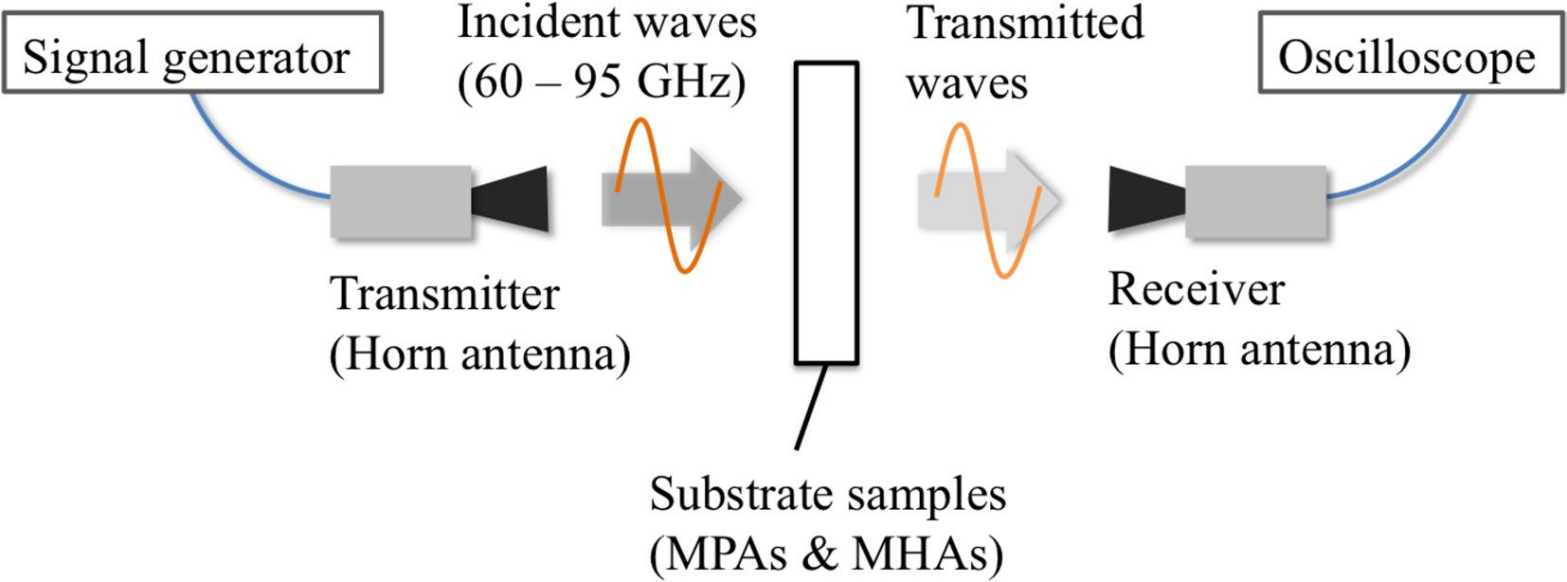
Measurement system.

To experimentally validate the situations described in Section “Basis of spoof surface plasmon polaritons generations on a metal plate array” experimentally, the measurement system shown in Fig. 16 was used. In this system, MPAs and MHAs, the pair of complimentary SSPP structures, are used as samples to show the breakdown of Babinet’s principle by comparing the transmission characteristics of the complementary structures. The experimental setup is very similar to that in our previous report^34,35^, which is briefly reviewed below. The horn antennae are used as a transmitter and a receiver. The transmitted signals, the frequencies of which vary from 60 GHz to 95 GHz continuously in time are detected by the oscilloscope as a time waveform which shows the frequency characteristics of the transmitted waves. By comparing the measured results in the cases with and without the sample, the transmission characteristics are obtained.

**Figure 17:**
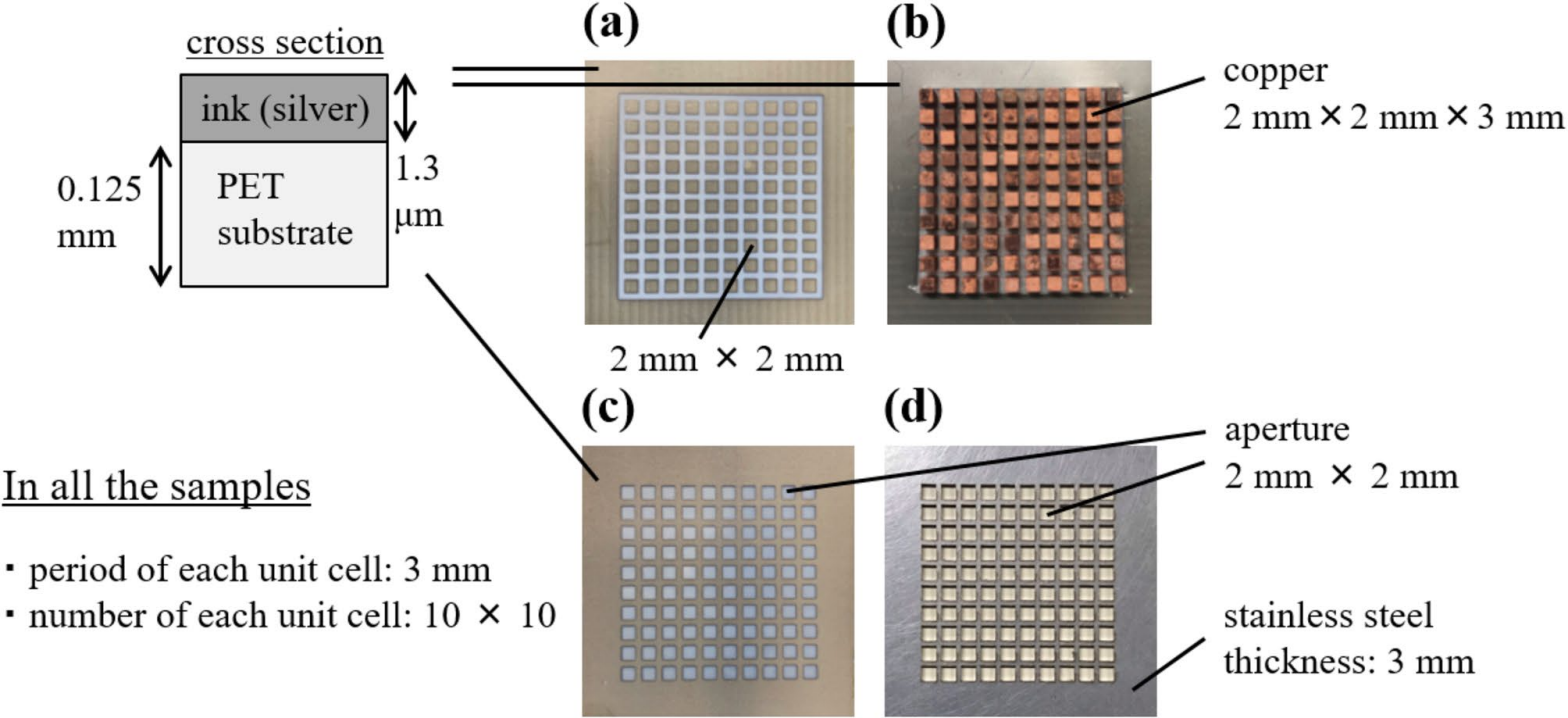
Experimental samples of MPAs and MHAs; (**a**) a sample of MPA with a thickness of $1.3 \,\mu \hbox {m}$ made of silver ink, (**b**) a sample of MPA with a thickness of 3 mm made of copper blocks, (**c**) a sample of MHA with a thickness of $1.3 \,\mu \hbox {m}$ made of silver ink, (**d**) a sample of MHA with a thickness of 3 mm made of stainless steel.

**Figure 18:**
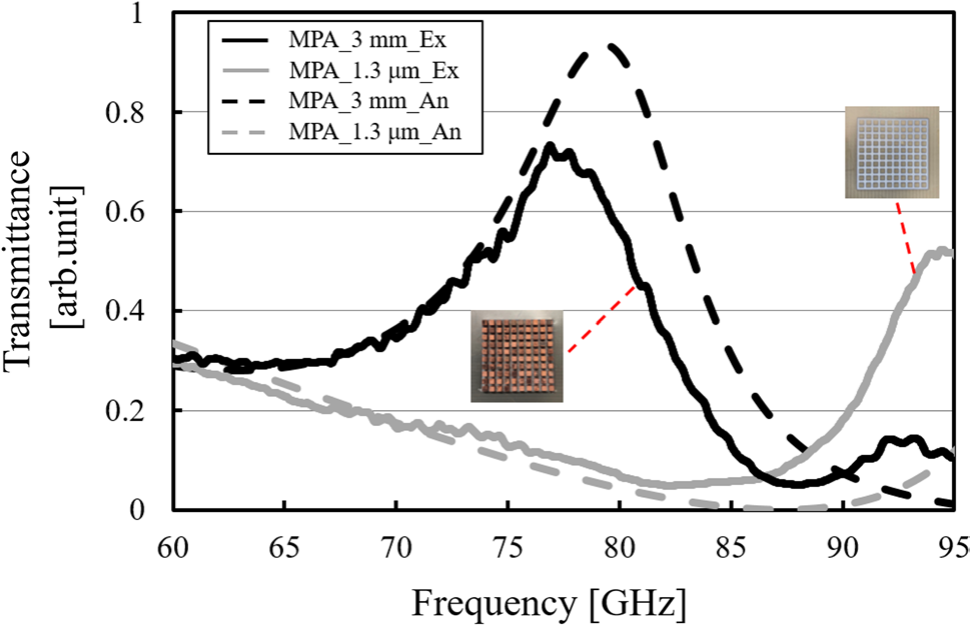
Thickness dependency of transmission characteristics in the thin and thick cases of the metal plate array (solid lines), compared to the corresponding analytical results (dotted lines).

**Figure 19:**
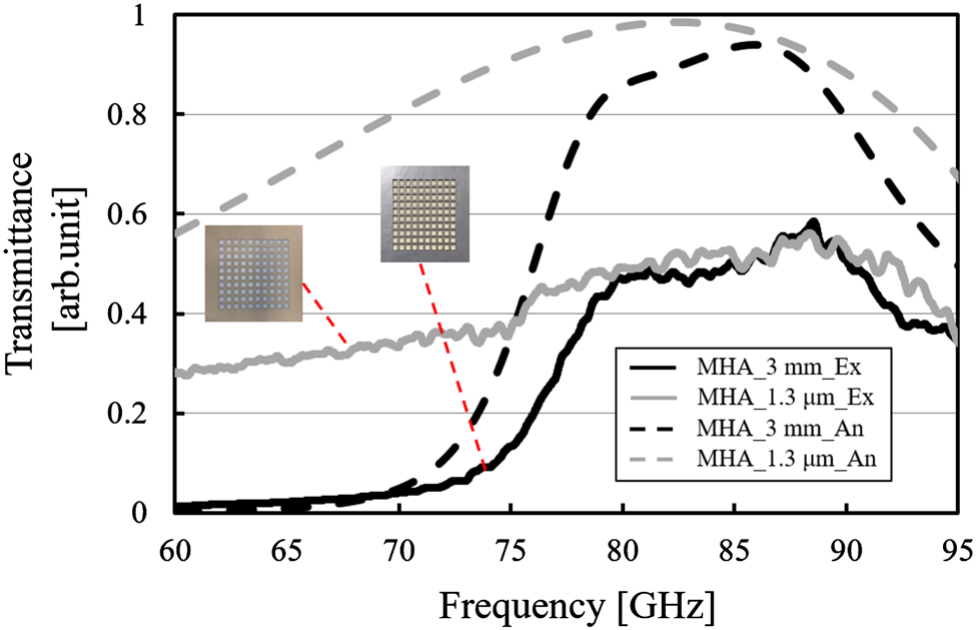
Thickness dependency of transmission characteristics in the thin and thick cases of the metal hole array (solid lines), compared to the corresponding analytical results (dotted lines).

The samples used in the experiments are shown in Fig. 17. Samples of (a) and (b) are the thin and thick MPAs on PET substrates, and the conducive parts of sample (a) are silver ink which is printed on the substrate. The thickness of the ink and the substrate are $1.3 \,\mu \hbox {m}$ and 0.125 mm. The unit cell of the conductor parts is a square ($2\, \hbox {mm} \times 2\, \hbox {mm}$) with a period of 3 mm. The unit cell of (b) has rectangular-shaped copper conductors ($2 \,\hbox {mm} \times 2\, \hbox {mm} \times 3\, \hbox {mm}$) that are arranged two-dimensionally with a period 3 mm, and the frame is made of the silver ink. Samples (c) and (d) are the thin and thick MHAs; (c) is also made of the silver ink, and its unit cell has a square aperture ($2 \,\hbox {mm} \times 2\, \hbox {mm}$) with a period of 3mm. Sample (d) is made of a stainless steel 3 mm thick, and its unit cell has a square aperture ($2 \,\hbox {mm} \times 2\, \hbox {mm}$) with period 3 mm. The experimental results with these MPAs and the MHAs are shown in Figures 18 and 19, respectively.

**Figure 20:**
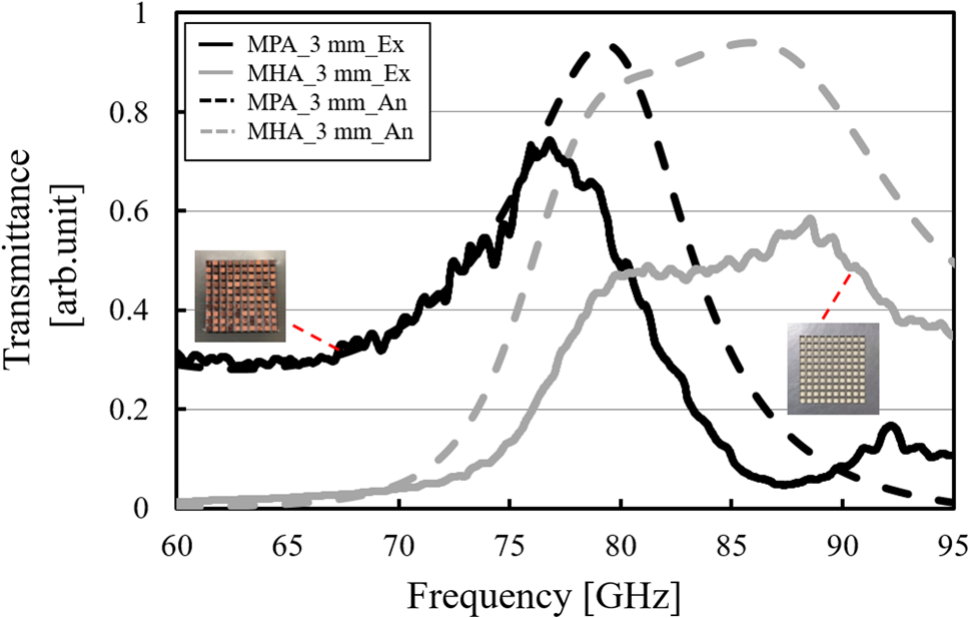
Experimental demonstration of the breakdown of Babinet’s principle using the complimentary structures (MPAs and MHAs) with a wavelength-size thickness.

In Fig. 18, the result confirms the difference of the transmission characteristics between the thin and thick MPAs. Consideration together with the analytical results shown in Fig. 9 shows that the thin MPA has a band-stop effect around 88 GHz, which originates in a functional impedance surface based on its equivalent circuit model. In contrast, the thick MPA has a band-pass effect around 77 GHz caused by the excitation of the SSPP modes. Here, since the analytical results are obtained by simulating the thin model with the corresponding substrate (PET), the experimental results are in good agreements with the analytical results in the tendency of frequency responses and the resonant frequencies and have high validities in terms of reproducibility. The overall transmittance values of the experimental results are considered to be lower than those of the analytical results due to the losses of the actual substrate.

In Fig. 19, as opposed to the MPA cases, the results show the invariance of the transmission characteristics with the band-pass effect between the thin and thick MHAs around 88 GHz in both cases. And they are also consistent with the corresponding analytical results including the electric distributions in Fig. 11 from the viewpoint of the tendency of the frequency responses and the resonant frequencies since the analytical results are obtained by using the MHA models with PET substrates as well as the above MPA cases. And the experimental results also have high validities in terms of reproducibility. Due to the same reason as the MPA case, the overall transmittance values of the experimental results become lower than those of the analytical results. Since the result of the thin case shows a band-pass effect, considered together with the above MPA results, this fact also supports the idea that the two thin models can be considered to be based on the impedance surface theory with lumped elements, and they follow Babinet’s principle. Also, as well as the above discussion of the thin case, the fact that the results of the two thick cases confirm band-pass effects also supports the idea that the two thick structures excite the resonant modes originating from each SSPP mode.

Finally, the breakdown of Babinet’s principle is demonstrated by the analytical and experimental results of the thick cases in Fig. 20. The results confirm that the complimentary structures (with the same 3 mm thickness) show the same transmission characteristics of band-pass effects. If the results are in accordance with the principle, the MPA results and the MHA results must respectively show opposite characteristics, such as band-pass and band-stop effects. This study provides the new physical insight that the principle, based on the symmetry of the physical behaviors of electric fields and magnetic fields, can be applied to the electromagnetic structure only when the complimentary structures used can be treated as boundaries. Furthermore, the breakdown indicates that SSPP modes can be more controllable than conventional since arranged conductive structures such as MPAs also can be excited the mode. And if the arranged structure is used, its band-pass feature of waves can be tuned flexibly by thickness so that required accuracy of the cross section-design becomes more relaxed. The fact has an important significance in industrial fabrication processes such as an artificial lens, an antenna, and a reflector based SSPP excitations.

## Conclusion

The breakdown of Babinet’s principle was discussed and examined with the use of complementary SSPP structures, MPAs and MHAs. First, SSPP mode formation on an MPA was introduced by theoretically deriving the effective relative permittivity with a Drude-type frequency response and the SSPP dispersion relation on an MPA following Pendry’s theory of SSPPs in the case of an MHA. In the electromagnetic numerical analyses, the thickness dependencies of the transmission characteristics in the case of MPAs and MHAs were discussed. The analytical results confirmed that the characteristics of the MPA changed from band-stop to band-pass as its thickness increased. On the other hand, with MHAs, all the cases showed band-pass features regardless of thickness. From the angular dependencies of the MPAs and the MHAs, the their resonant mechanisms in the thin and thick cases were considered to be based on an impedance surface model with lumped elements used for frequency selective surfaces (FSSs) and the SSPP theory, respectively. In the experiments, thin and thick models of MPAs and MHAs with the same size as the analytical models were provided and their transmission characteristics investigated. The results also showed the same tendencies as the analytical results. Finally, the breakdown of Babinet’s principle was demonstrated experimentally by using the thick models of the MPA and the MHA, and this fact gives us the new physical insight that the principle can be applied to the electromagnetic structure only on the premise that the complimentary structures used can be treated as boundaries.
